# Hydrothorax

**Published:** 1874-05

**Authors:** C. P. Allen


*THE PHILADELPHIA MEDICAL TIMES—February*, 1874.

Dropsy of Left Pleura Cured by Laxatives and Digitalis.—*By C. P. Allen, M.D.*—The above case is interesting in at least two things. First, the insidious manner in which the disease made its apperance, and the amount of effusion without pain; second, the ready manner in which it yielded to gentle laxatives and digitalis.

I have always found it difficult to remove effusions in closed cavities and cysts by means of laxatives and diuretics, or at best the process is a very slow one.

In a case of large effusion in the pleura, it is very readily understood why it is so. The lung is greatly compressed by the fluid, so that little or no air enters it, and the blood-vessels are in a congested condition. The sound lung is more or less congested and compressed by the over-distension of the opposite pleura, and it is well known that this condition is unfavorable to absorption. Another reason is that coagulable lymph is very apt to coat over the pleura, and render absorption slow and difficult.

In the above case, the probability is that very little lymph had been effused, the inflammation having been so mild in its character, and that the laxative given relieved the bowels and portal system by a direct action, and the blood-vessels of the chest by a secondary action, thus favoring the effect of the digitalis as a diuretic and the accompanying relief of the effusion.

We have often found very marked relief from the use of digitalis in anasarca of the legs, with or without effusion in the peritoneal cavity, occurring in elderly people having a quick and feeble pulse, with atony or enlargement, and dilatation, of the heart.

